# Adolescent idiopathic scoliosis without limb weakness: a differential diagnosis of core myopathy?

**DOI:** 10.1186/s12891-015-0629-8

**Published:** 2015-08-05

**Authors:** Rafael de Paiva Luciano, Eduardo Barros Puertas, Delio Eulalio Martins, Flavio Faloppa, David Del Curto, Luciano Miller Reis Rodrigues, Beny Schmidt, Acary Souza Bulle de Oliveira, Marcelo Wajchenberg

**Affiliations:** Universidade Federal de Sao Paulo - Brazil – R. Borges Lagoa, 783, 5 andar, Vila Clementino, SP Zip Code - 04038-031 Brazil

## Abstract

**Background:**

Core myopathies are a clinically and genetically heterogeneous group of congenital myopathies with the common defined histopathological feature of focally reduced oxidative activity on muscle biopsy. It has a low incidence, however, recent articles show broad clinical spectrum, suggesting that the real incidence should be considerably larger than previously described. Due to the important association between scoliosis and paravertebral muscle imbalance, numerous authors study, by biopsy of the spinal rotator muscles, potential changes that may elucidate the etiology of adolescent idiopathic scoliosis.

**Case presentation:**

Two patients have been followed at Spine Group of Department of Orthopedics at Federal University of São Paulo, with an initial diagnosis of idiopathic scoliosis. Both patients had clinical and radiological findings compatible with it. The patients authorized, through the Term of Consent, intraoperative biopsy of muscle multifidus from the apex of the thoracic curve on concave and convex sides. After muscle biopsy was performed a histopathological analysis. As regard to the histopathological features: in both patients were identified, the presence of core structures in extensive areas with reduced oxidative activity running along the muscle fiber.

**Conclusions:**

All patients with ‘idiopathic’ scoliosis deserve a careful neurological evaluation, even if they have minimal muscle symptoms in the extremities. The frequent occurrence of scoliosis in patients with CORE Myopathies, supports the thesis that the change in the paravertebral muscle fiber must be the underlying pathogenic factor in scoliosis and may help us understand the onset and progression of curves in patients previously diagnosed with idiopathic scoliosis.

## Background

Scoliosis is a common complication present in various neuromuscular diseases that develops as a result of progressive muscle weakness in the paraspinal musculature [1]. Neuromuscular scoliosis typically appears during advanced stages of disease, and this form is progressive and associated with deformities of the sagittal plane [2]. However, the most common form of this deviation is adolescent idiopathic scoliosis (AIS) which is a tridimensional deformity, defined as lateral deviation of the spine associated to vertebral rotation, with also sagittal plane implication. It occurs without a known cause in adolescents who do not exhibit neurological or muscular disorders or other diseases [3–5].

Core myopathy constitutes a group of congenital myopathies that present histopathologic features of focal reduced oxidative activity in muscle biopsies and they are clinically and genetically heterogeneous [6–8]. Core myopathy was initially described by Magee and Shy in 1956 as having a low incidence, although recent studies have shown a broader clinical spectrum for this condition, which suggests that the real incidence is considerably higher than previously described [9–11].

We evaluated two patients with a diagnosis of AIS by collecting muscle multifidus biopsies during the surgical correction of their scoliosis deformities. During the histopathologic evaluation of these muscle fragments, we noted the presence of multiple core structures indicative of congenital core myopathies [6].

The clinical presentation of core myopathies can be widely variable, although most patients develop hypotonia or delayed motor development in early childhood. More severe presentations, such as fetal akinesia, as well as milder clinical scenarios of adult onset have also been described as manifestations of these myopathies. Furthermore, orthopedic complications such as scoliosis, congenital hip dysplasia, foot deformity, ligamentous laxity and patellar instability have also been associated with congenital myopathies [6]. The only clinical manifestation found in patients was the scoliosis. They demonstrated motor and neurological development adequate for their age. However, the presence of core myopathy symptoms without any associated weakness is not sufficient for a diagnosis of core myopathy [5].

The presence of progressive scoliosis in the two patients discussed here could be associated to the weakness of the paraspinal musculature. This weakness would be result of a mild form of myopathy or related to physiopathology of the AIS. The musculature analyzed was the multifidus, that arise from mammillary processes and pass to the spinous processes two to four level rostrad. Its main function is to act as agonist of the rotational movement of the spine [12]. Thus its dysfunction could cause a rotational deformity, characteristic of AIS.

Of the known causative factors for the development of core myopathy, genetic factors seem to be most important, as mutations in the skeletal muscle RYR1 gene and less frequently in the SEPN1 gene contribute to disease development. The genetic analysis of the patients was not performed because initially the muscle disease was not suspected. After examination of biopsies and the central CORE histopathological finding, the next step will be to analyze the genetic code. However, this may require time due to the size of the mutations associated to the central CORE. It is important to explain that biopsies are routinely performed due to a research protocol of the institution in order to investigate the role of muscle balance in adolescent idiopathic scoliosis. The patients in this report had no relatives with deformities or altered motor development.

Previous studies have demonstrated a clear association between RYR1 gene mutations and susceptibility to malignant hyperthermia, which is a pharmacogenetic predisposition to potentially deadly adverse reactions that occur in response to volatile anesthetics and muscle relaxants [13–15]. This susceptibility is of particular interest to spine surgeons, as it can lead to progressive idiopathic scoliosis and require surgical treatment.

Because of the important association between scoliosis and paravertebral muscle imbalance, numerous studies have evaluated biopsies of the spinal rotator muscles for potential changes that may elucidate the etiology of AIS [5, 16–23]. Here we describe two patients with clinical and radiological findings of AIS who were operated on and subjected to multifidus muscle biopsy, which demonstrated anatomopathological results suggestive of core myopathy.

## Case presentation

### Case 1

**Fig. 1:**
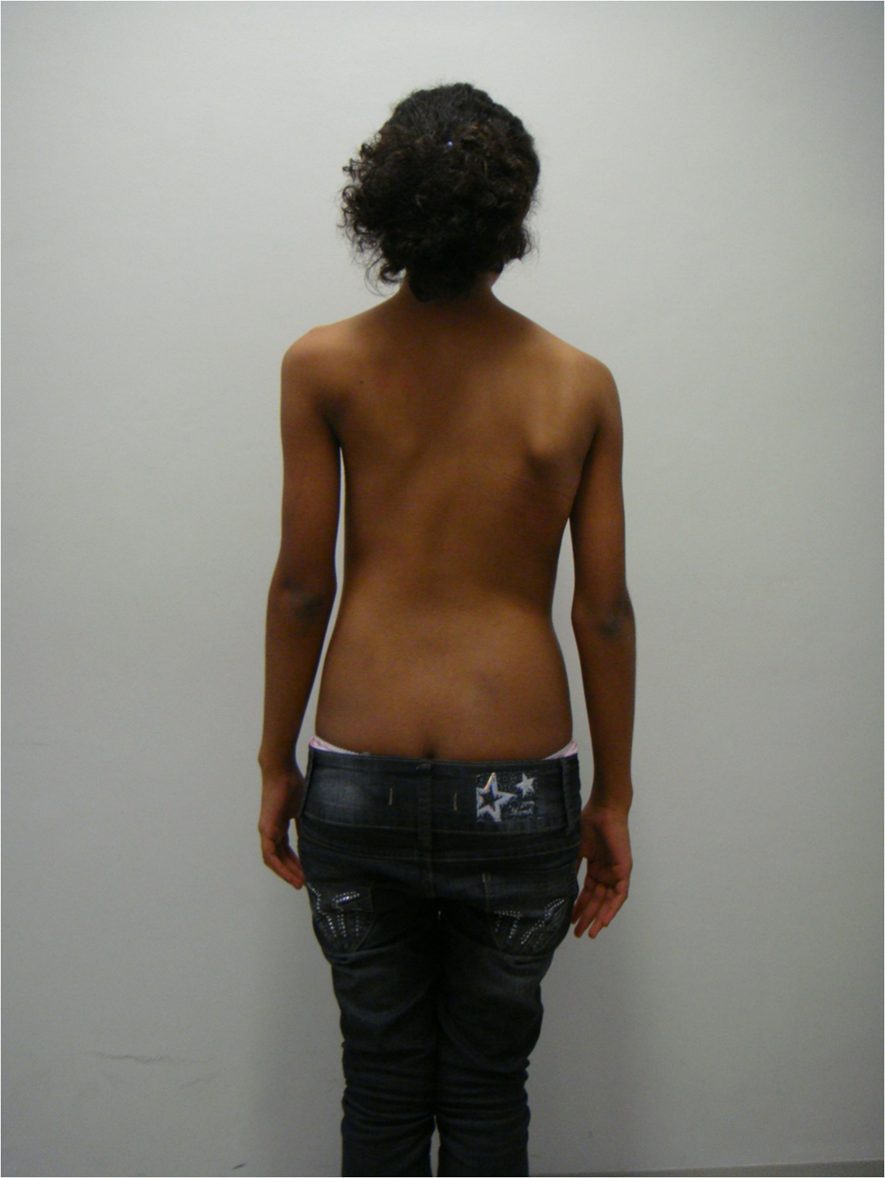
Case 1: The patient’s clinical aspect prior to surgery. Posterior view in orthostasis

**Fig. 2:**
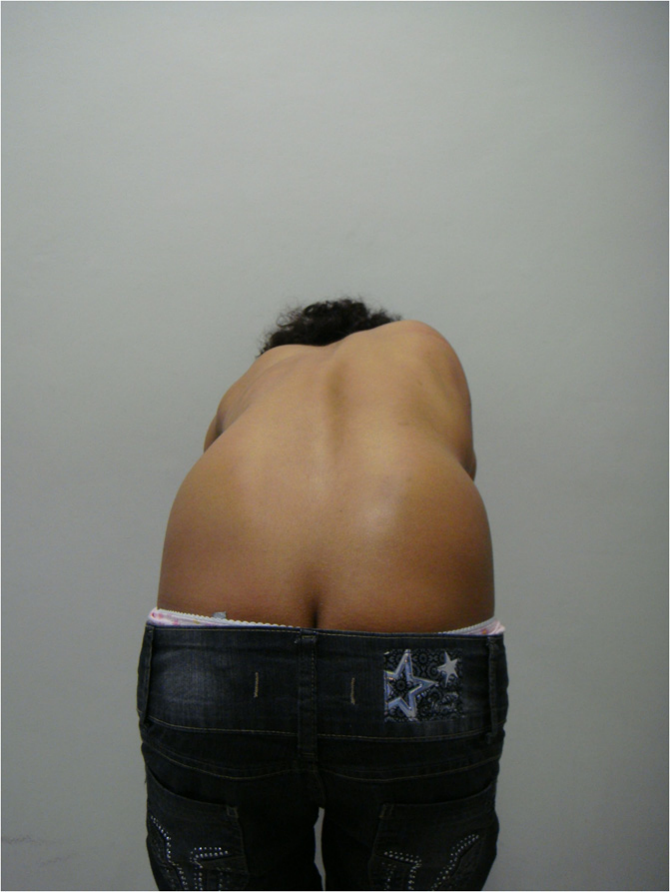
Case 1: The patient’s clinical aspect prior to surgery. Posterior view after Adam's Forward Bend Test

**Fig. 3:**
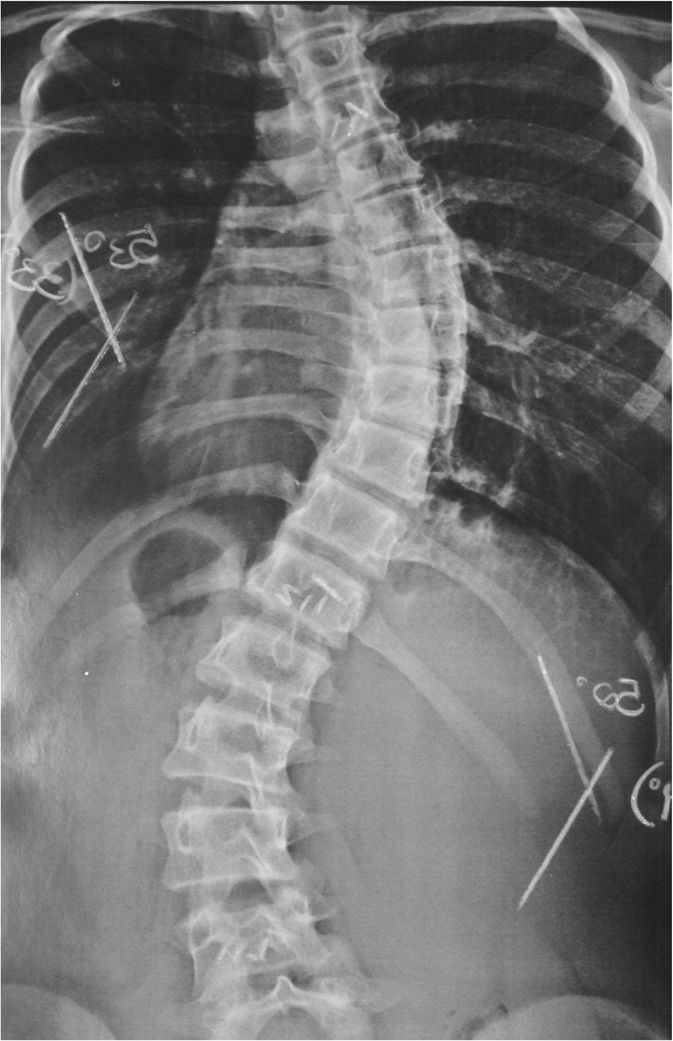
Case 1: Radiological aspect of the scoliosis - AP view

**Fig. 4:**
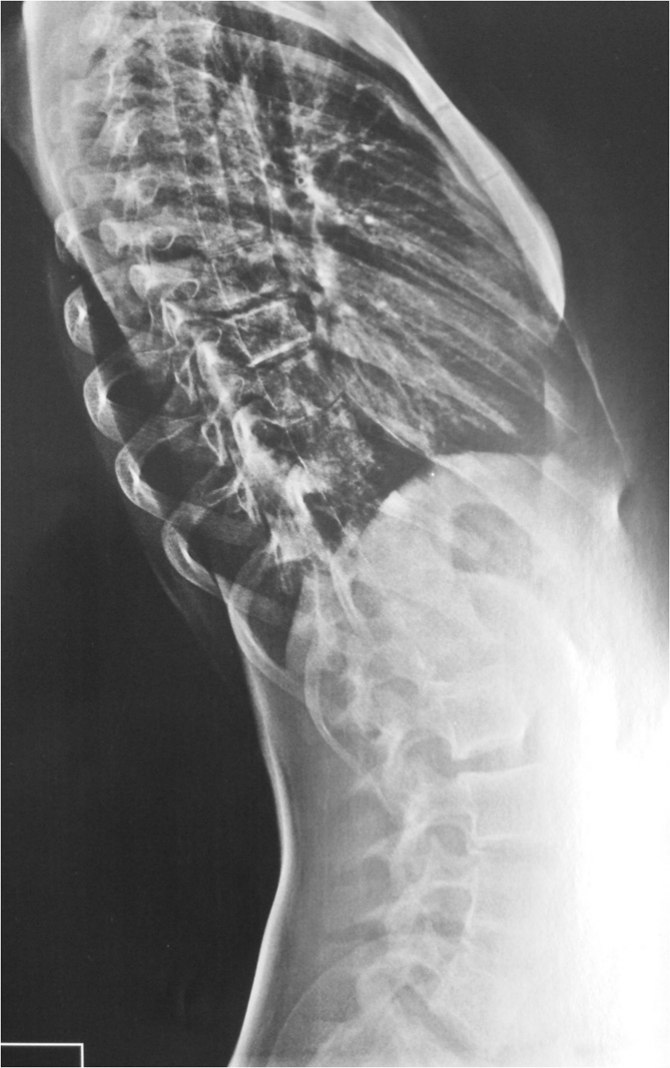
Case 1: Radiological aspect of the scoliosis – Perfil view

This patient, who was referred to as TTF, was 12 years old, female and of a mixed background. Scoliosis was first observed at age 10, but no other family members were affected. This patient had not yet reached menarche and demonstrated adequate neurological and motor development without any co-morbidities. The patient also demonstrated normal findings on a neurological examination. A physical examination did not detect skin changes, although the Adams maneuver revealed a thoracic right hump (Figs. 1 and 2). The scoliosis was classified as type III, according to the King and Lenke 1C classification, with a right thoracic curve of 53° and a left lumbar curve of 52° (Figs. 3 and 4).

The patient was monitored at the Outpatient Clinic of the Spine Group at Hospital Sao Paulo of the Federal University of São Paulo for two years and received surgery on October 31, 2011, Figs. 5 and 6.

**Fig. 5:**
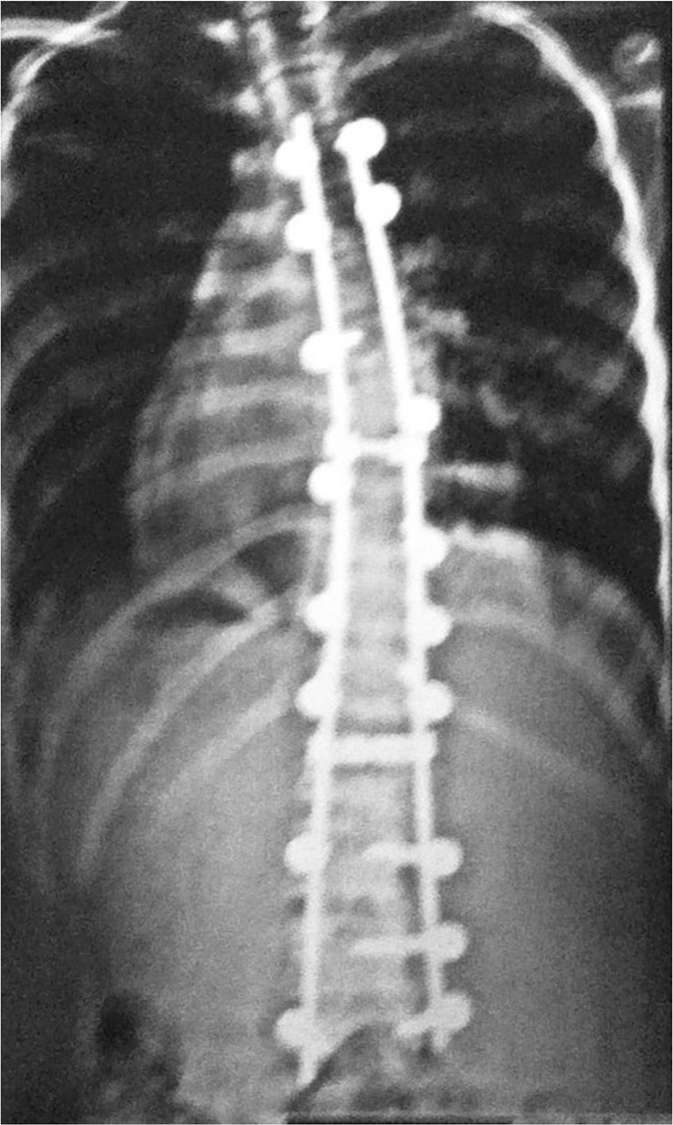
Case 1: The patient after surgery – Radiograph in AP view

**Fig. 6:**
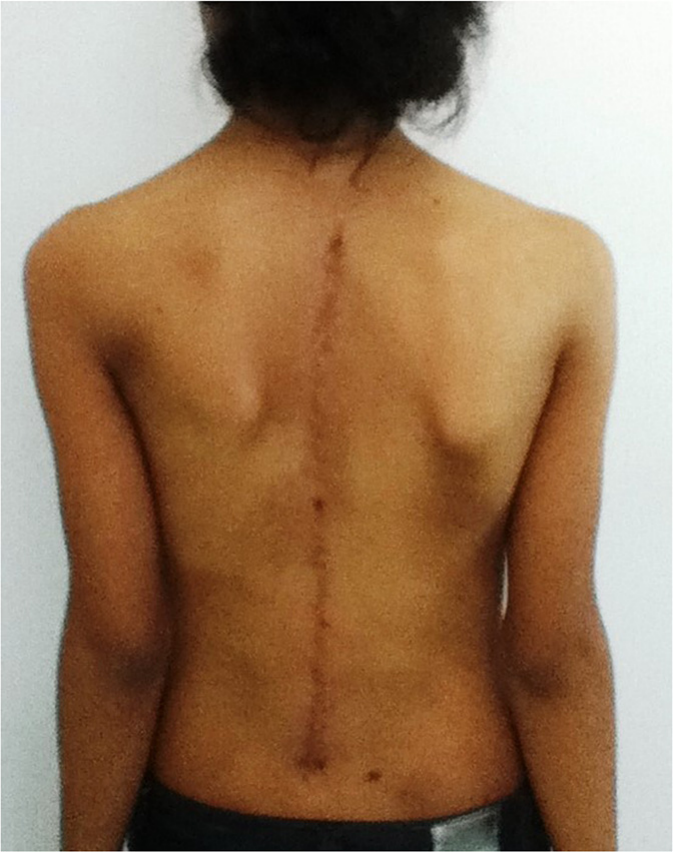
Case 1: The patient after surgery – Clinical aspect

### Case 2

**Fig. 7:**
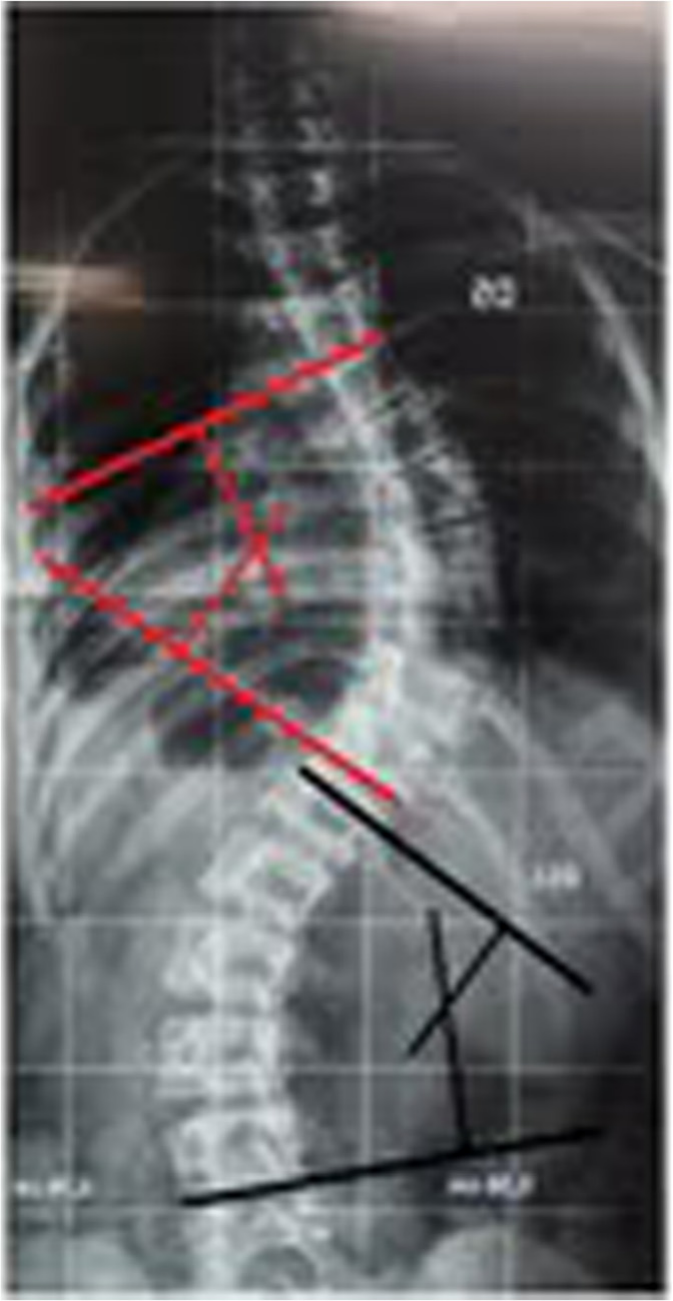
Case 2: Radiological aspect of the scoliosis - AP view

**Fig. 8:**
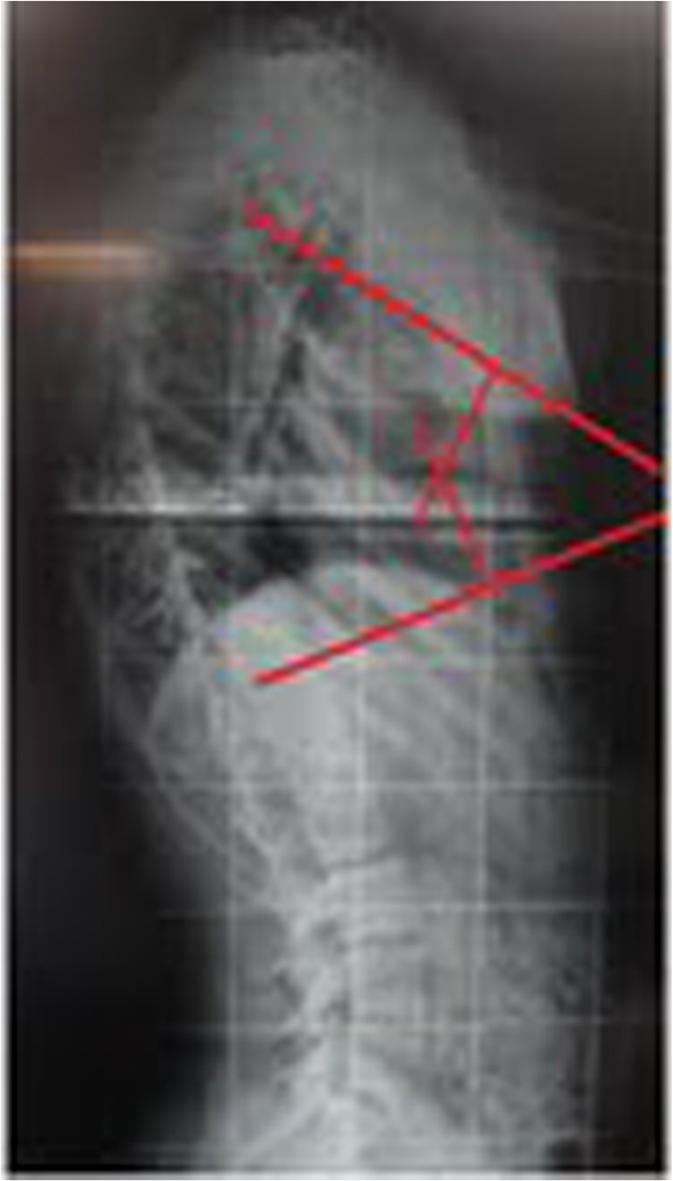
Case 2: Radiological aspect of the scoliosis – Perfil

This patient, who was referred to as CYMH, was 12 years and 10 months of age, oriental and female. She reached menarche at 12 years and 2 months of age and demonstrated adequate neurological and motor development without any co-morbidities. Scoliosis was noted at 10 years of age. A physical examination showed no cutaneous alterations, the neurological examination was normal, and the Adams maneuver indicated a right thoracic hump. The scoliosis was classified as type II, according to the King classification system, and presented as a right thoracolumbar curve (T5-T11) of 62°, a left lumbar curve (T12-L4) of 55° and kyphosis (T5-T12) of 50° (Figs. 7 and 8)). The patient was monitored at the Outpatient Clinic of the Spine Group at Hospital Sao Paulo of the Federal University of São Paulo for 2 years and received surgery on 02/07/2012, Figs. 9 and 10.

**Fig. 9:**
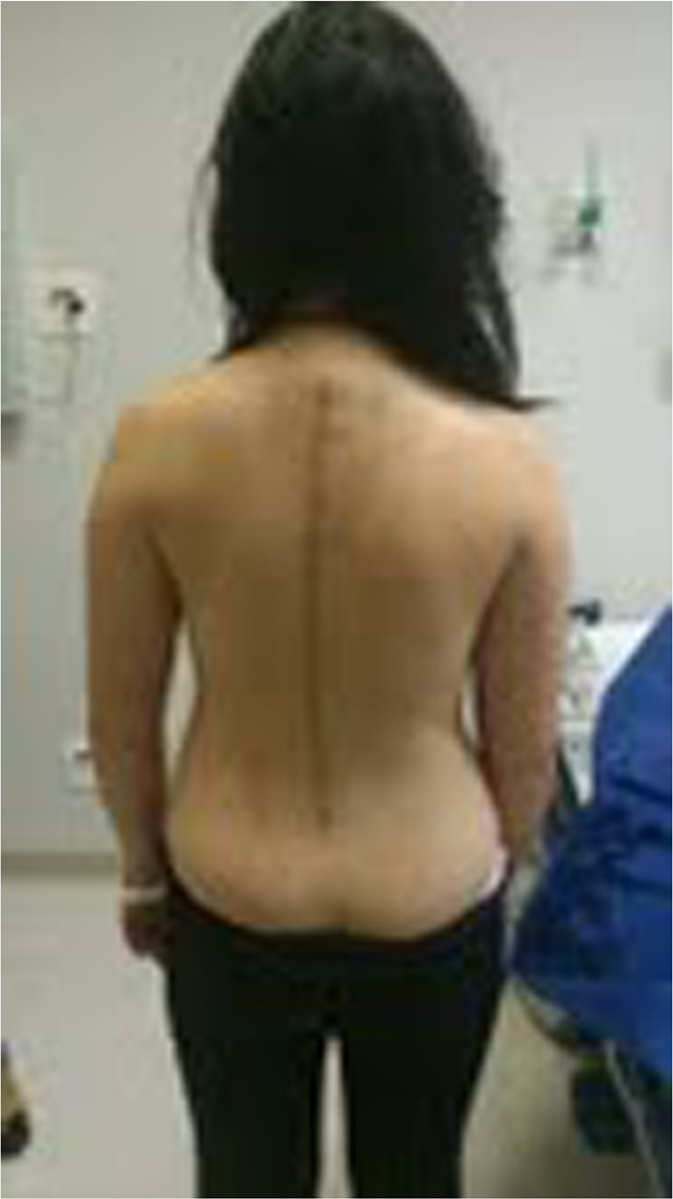
Case 2: The patient after surgery – Radiograph in AP view

**Fig. 10:**
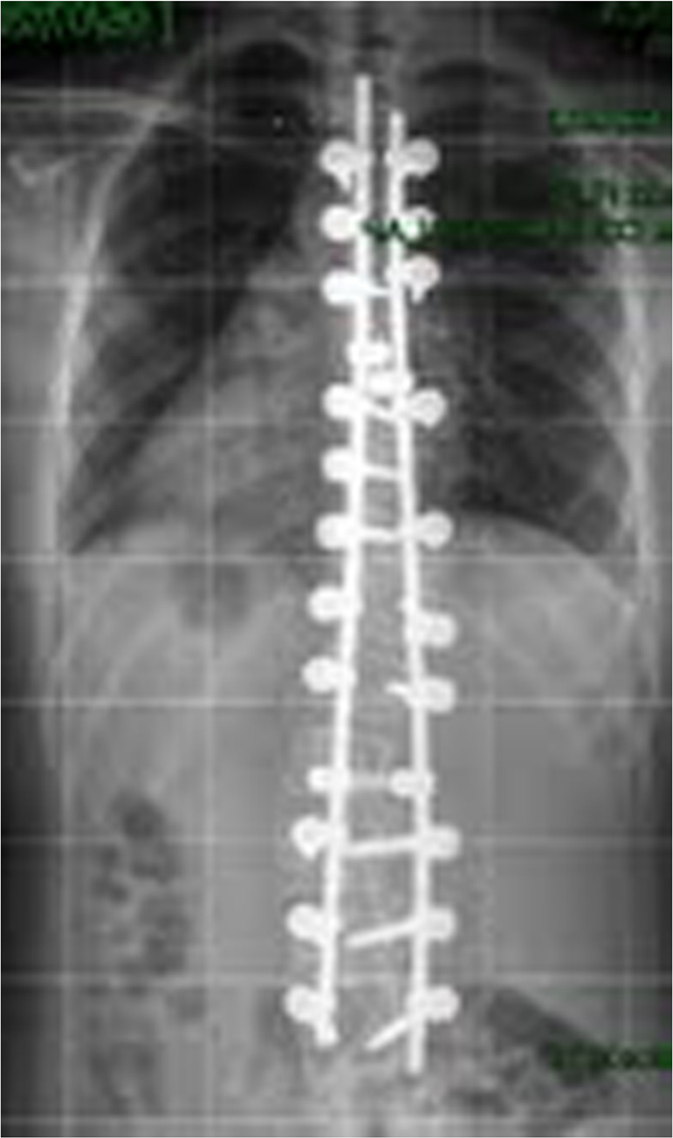
Case 2: The patient after surgery – Clinical aspect

**Fig. 11:**
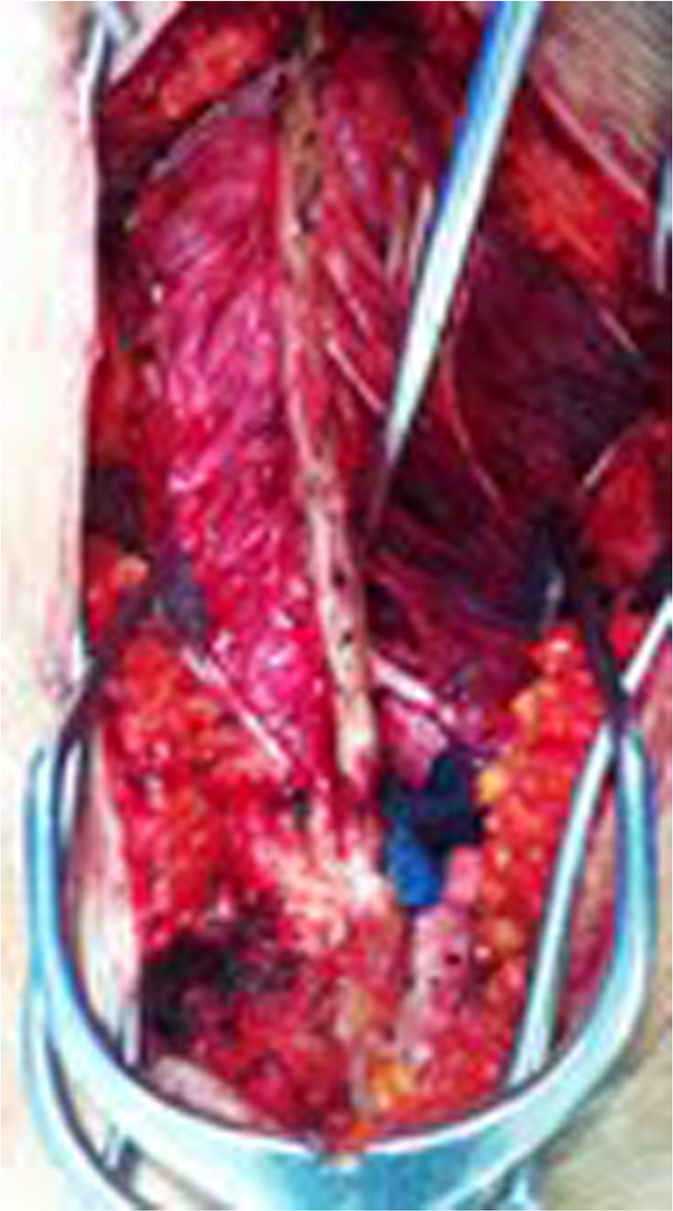
Muscle biopsy of rotator muscles

Both patients families provided informed consent for intraoperative multifidus muscle biopsy at the apex of the thoracic curve on the concave and convex sides (Fig. 11). The parents/guardians of both patients gave consent for the publication of the case report and the pictures. Muscle biopsies from patients diagnosed with scoliosis are performed at the Hospital São Paulo after approval from the Ethics Committee at the Federal University of São Paulo, for Histopathological Analysis in the Laboratory of Neuromuscle Diseases of the Department of Neurology and Neurosurgery of the Federal University of Sao Paulo.

### Histopathological evaluation

**Fig. 12:**
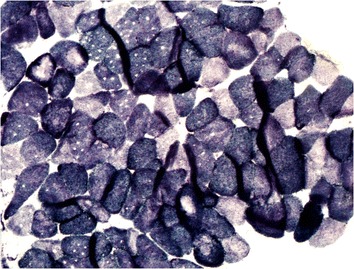
Patient 1: Serial transverse sections of a biopsy from the multifidus muscle activity in many fibers. NADH × 100 – Single prominent cores devoid of oxidative

**Fig. 13:**
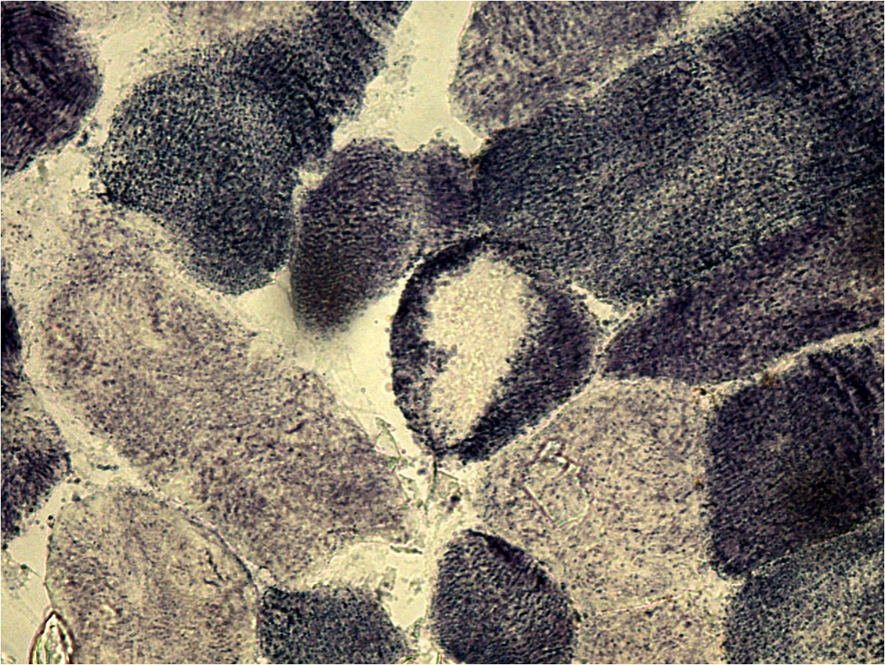
Patient 1: Serial transverse sections of a biopsy from the multifidus muscle activity in many fibers. SDH × 400 – The core is clearly delimited from the surrounding cytoplasm

**Fig. 14:**
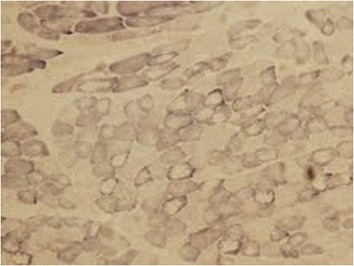
Patient 2: Serial transverse sections of a biopsy from the multifidus muscle. SDH × 100 – Single prominent cores devoid of oxidative activity

**Fig. 15:**
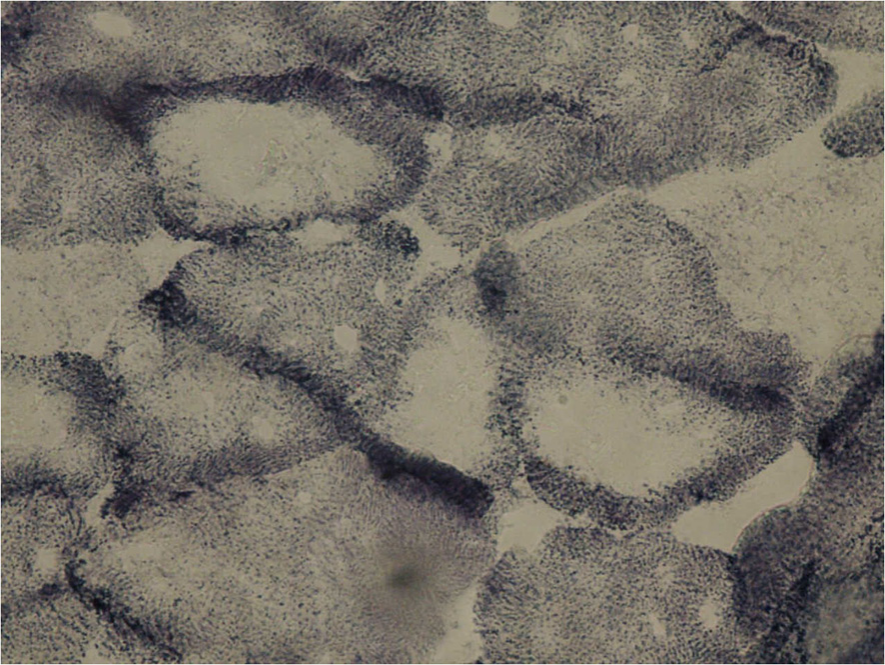
Patient 2: Serial transverse sections of a biopsy from the multifidus. muscle. SDH × 400 – The core is clearly delimited by the surrounding cytoplasm

The collection of muscle samples were performed according to the methods described by Schmidt et al. [24], whereby the extracted pieces were stored in a polystyrene box with ice and protected by a gauze pad. The material was immediately taken to the laboratory, where the fragments were removed and placed on a cork, fixed with tragacanth gum and covered with common talc. The set was then immersed in liquid nitrogen at −180 °C for 20 s, and the blocks were stored at −80 °C. Serial sections were cut using a cryostat at −22 °C (Figs. 12, 13, 14 and 15).

Samples were analyzed for fiber type, muscular atrophy and hypertrophy, fatty proliferation, fibrosis, presence of hyaline fibers, mitochondrial proliferation, muscular necrosis, nuclear centralization, presence of central core myopathy and inflammation (Table 1).

**Table 1:** Histopathological alterations in the paraspinal skeletal muscle of patients with adolescent idiopathic scoliosis

CASE 1	Necrosis	Atrophy	Hypertrophy	Fatty proliferation	Fibrosis	Inflammation
Concave	Moderate	Moderate	Mild	Moderate	Moderate	Presence
Convex	Mild	Mild	Scarce	Absent	Scarce	Absent
CASE 2						
Concave	Scarce	Scarce	Absent	Scarce	Scarce	Absent
Convex	Scarce	Mild	Scarce	Moderate	Scarce	Absent

The muscle fiber type I was prevalent in the biopsies. In both sides of the curve observed the presence of central core. The isolated description of these cases does not allow the comparison of the changes observed between the two sides of the curve. This analysis will be further after biopsies of a larger number of patients.

## Conclusions

Regardless of clinical outcome, the reported changes in the patients may help to understand the real influence of the rotator muscles of the spine in the etiology and perpetuation of “idiopathic” scoliosis adolescents. It is necessary further studies with histopathological evaluation of paravertebral muscles in a larger number of patients to elucidate the role of muscle in the etiology and pathophysiology of AIS. However, if genetic analysis of these patients will be positive for genes related to myopathy CORE, these patients developed a secondary scoliosis and, therefore, they can’t be classified as idiopathic scoliosis.

## Consent

Written informed consent was obtained from both the patient and guardians for publication of this Case report and any accompanying images. A copy of the written consent is available for review by the Editor of this journal.

## Abbreviations

AIS: Adolescent idiopathic scoliosis
Fig: Figure
